# Highly efficient ZnO/WO_3_ nanocomposites towards photocatalytic gold recovery from industrial cyanide-based gold plating wastewater

**DOI:** 10.1038/s41598-023-49982-6

**Published:** 2023-12-20

**Authors:** Satjaporn Sangkhanak, Naphaphan Kunthakudee, Mali Hunsom, Prakorn Ramakul, Karn Serivalsatit, Kejvalee Pruksathorn

**Affiliations:** 1 Department of Chemical Technology, Faculty of Science, Chulalongkorn University, Phayathai Road, Pathumwan, Bangkok, 10330 Thailand; 2 Department of Chemical Engineering, Faculty of Engineering, Mahidol University, Phuttamonthon 4 Road, Nakhon Pathom, 73170 Thailand; 3 Associate Fellow of Royal Society of Thailand (AFRST), Bangkok, 10300 Thailand; 4 Department of Chemical Engineering, Faculty of Engineering and Industrial Technology, Silpakorn University, Nakhon Pathom, 73000 Thailand; 5 Department of Materials Science, Faculty of Science, Chulalongkorn University, Phayathai Road, Pathumwan, Bangkok, 10330 Thailand; 6 Photocatalysts for Clean Environment and Energy Research Unit, Faculty of Science, Chulalongkorn University, Bangkok, 10330 Thailand

**Keywords:** Materials science, Nanoscience and technology, Optics and photonics

## Abstract

Discharging the gold-contained wastewater is an economic loss. In this work, a set of ZnO/WO_3_ was facile synthesized by hydrothermal method in order to recover gold from the industrial cyanide-based gold plating wastewater by photocatalytic process. Effect of ZnO contents coupled with WO_3_ was first explored. Then, effects of operating condition including initial pH of wastewater, type of hole scavenger, concentration of the best hole scavenger and photocatalyst dose were explored. A series of experimental results demonstrated that the ZnO/WO_3_ nanocomposite with 5 wt% ZnO (Z_5.0_/WO_3_) depicted the highest photocatalytic activity for gold recovery due to the synergetic effect of oxygen vacancies, a well-constructed ZnO/WO_3_ heterostructure and an appropriate band position alignment with respect to the redox potentials of [Au(CN)_2_]^−^ and hole scavengers. Via this ZnO/WO_3_ nanocomposite, approximately 99.5% of gold ions was recovered within 5 h using light intensity of 3.57 mW/cm^2^, catalyst dose of 2.0 g/L, ethanol concentration of 20 vol% and initial pH of wastewater of 11.2. In addition, high stability and reusability were observed with the best nanocomposite even at the 5th reuse. This work provides the guidance and pave the way for designing the ZnO/WO_3_ nanocomposite for precious metal recovery from a real industrial wastewater.

## Introduction

Based on the unique properties considered in terms of high electrical conductivity, reliability and high corrosion resistance, gold is often used as the part for connectors, wiring pads, conductors, electrodes or passive components to provide a corrosion resistance electric layer on the metal substrates^1^. The deposition of gold on substrates can be conducted by either electroplating or electroless plating via the cyanide-based plating bath or non-cyanide-based plating^2^. Despite the utilization of toxic chemicals and emission of toxic wastewater which requires a proper management system, the cyanide-based gold plating is still widely used because it gives a stable and capable gold films with high corrosion resistance, high electrical and thermal conductivities, and high ductility^3,4^. Due to an increasing demand for electronic devices/equipment during this digital era, a number of electronic components are manufactured to serve the need of electronic markets^5^. In this circumstance, a high quantity of wastewater containing the gold-cyanocomplexes is generated. Therefore, several processes have been developed and conducted to treat the metal-cyanocomplexes containing wastewater, such as electrolysis^6^, ion exchange^7^, chemical or advanced chemical oxidations^8,9^, solvent extraction^10,11^, and adsorption^12,13^. However, their actual application is strictly restricted by their specific drawbacks. For example, the electrolysis is the energy intensive process with low metal selectivity, while the ion exchange process is costly and requires the precise control^14^. Both adsorption and chemical oxidation usually produce the waste product or sludge, thus requiring the downstream process to complete the waste treatment^14^.

Besides all mentioned processes, the photocatalytic process is another promising process that can simultaneously remove free cyanide and recover gold ions in metallic form, which can be reused in subsequent processes. As brief, the photocatalytic process is a chemical-based process that can be proceeded via the joint operation between appropriate intensive light and semiconductor (photocatalyst)^15^. When the photocatalyst (S) is irradiated by the suitable light, the electrons will transfer from valance band (VB) to conduction band (CB), leaving the photogenerated holes (reaction (R1))^16,17^. The contained [Au(CN)_2_]^−^ species can readily react with the photogenerated electrons at CB, yielding the metallic gold deposited on the surface of photocatalyst and free cyanide (CN^−^) (reaction (R2))^18^. Meanwhile, the contained hydroxide ions (OH^−^) can react with the photogenerated holes to produce the hydroxyl radicals (OH^·^) (Reaction (R3)). Subsequently, the formed OH^·^ can consecutively react with the released free cyanide ions (CN^−^), yielding the cyanate species (OCN^−^) as the main product (reaction (R4))

R1 $${\text{S}}\mathop{\longrightarrow}\limits^{hv}{\text{e}}^{ - } + {\text{ h}}^{ + }$$

R2 $$\left[ {{\text{Au}}\left( {{\text{CN}}} \right)_{{2}} } \right]^{ - } + {\text{ e}}^{ - } \to {\text{Au }} + {\text{ 2CN}}^{ - }$$

R3 $${\text{OH}}^{ - } + {\text{ h}}^{ + } \to {\text{OH}}^{\cdot}$$

R4 $${\text{CN}}^{ - } + {\text{ 2OH}}^{\cdot} \to {\text{OCN}}^{ - } + {\text{ H}}_{{2}} {\text{O}}$$

Based on the proposed mechanism, it can be noticed that the photocatalytic reduction of [Au(CN)_2_]^−^ can well proceed in the basic environment. Nevertheless, the reduction reaction of [Au(CN)_2_]^−^ is still sluggish due to its high negative reduction potential (− 0.57 V/NHE^19^), thus limiting the use of variety photocatalysts. That is, only the photocatalysts that have a more positive VB position than the oxidation potential of OH^−^ and also have a more negative CB position than the reduction potential of [Au(CN)_2_]^−^ can complete the photocatalytic gold recovery from the metal-cyanocomplexes contained solutions such as ZnO^20,21^, ZnS^22^, TiO_2_ and TiO_2_-based materials^23,24^. Among all explored photocatalysts, it is noteworthy that TiO_2_ or TiO_2_-based materials exhibited the best photocatalytic activity, probably due to its good surface property and high thermal-chemical resistance^25,26^. Nevertheless, the popular used materials have a considerably high cost, which is not practical in actual operation. Therefore, the development of cheap and efficient photocatalyst for gold recovery is a key issue in this circumstance.

Tungsten trioxide (WO_3_) is an attractive semiconductor for the photocatalytic applications, owing to its non-toxic, biocompatibility, inexpensive, bandgap tunability, high electron storage capability and high stability in violent conditions^27,28^. Besides, it possesses a narrow bandgap (2.4–2.8 eV), which can theoretically absorb almost 12% of solar light^29^. Nevertheless, it still has a poor photocatalytic activity due to its low kinetics, sluggish charge transfer, high recombination rate of photogenerated charge carriers^29,30^. To overcome these drawbacks, many strategies have been carried out to improve the photocatalytic activity of WO_3_ by structural modification, co-catalyst hybridization, metal doping and heterojunction^29–32^. Among all developed strategies, the hybrid heterojunction is recognized as the most effective strategy to improve the photocatalytic performance of WO_3_ because of the formation of a more negative CB, which can suppress the rate of charge recombination^29^. Several materials have been selected to hybrid with WO_3_ such as Ag_3_PO_4_, AgI, TiO_2_, RGO, g-C_3_N_4_, Bi_2_WO_6_^30,32^.

Hybridizing WO_3_ with ZnO is currently gaining attention due to the improvement of surface acidity of resultant nanocomposites and visible light absorption as well as the improved photocatalytic activity of the single counterpart^28^. Nevertheless, the ZnO/WO_3_ nanocomposites were often applied for the photocatalytic degradation of pollutants. For examples, the ZnO/WO_3_ nanocomposite synthesized by hydrothermal method displayed a high charge separation and a redshift of light absorption compared with the counterpart semiconductors due to W–O–Zn linkage formation, which can promote a high photocatalytic degradation of methyl orange (MO)^33^. The ZnO-WO_3_ synthesized by ultrasound-assisted synthesis exhibited a great visible light response as well as the degradation of NO_*x*_, approximately twice as high as that of conventional composites^34^. The ZnO/WO_3_ photocatalyst synthesized by a calcination method induced the formation of the immanent electric field between both semiconductors, which can drive the Z-scheme charge transfer mechanism for photocatalytic degradation of MO, rhodamine B (RhB) and bisphenol A (BPA)^35^. The addition of ZnO on WO_3_ induced by the Pechini sol–gel method induced the creation of a W–O–Zn bond, which can enhance the UV–Vis light absorption, reduce the rate of charge recombination, increase the specific surface area and promote the photocatalytic desulfurization of thiophene^36^. The ZnO/WO_3_ photocatalyst synthesized by hydrothermal and calcination methods exhibited the outstanding photocatalytic H_2_O_2_ production due to the generation of interfacial internal electric field (IEF) in the S-scheme heterojunction, which can further suppress the charge recombination and empower the electrons to participate the photocatalytic reaction^37^. A ZnO@WO_3_ nanocomposite synthesized by the hydrothermal method exhibited a short rod-like structure with a long lifespan of charge carriers and a low charge transfer resistance^38^. Besides, hybridizing WO_3_ with ZnO importantly changed the forbidden band width of the nanocomposites, thus improving the visible light response and the photocatalytic degradation of RhB. The WO_3_/ZnO composite synthesized by the co-precipitation and ultra-sonication routes exhibited the synergetic effect of counterpart semiconductors to increase the charge separation and reduce the charge recombination, which can remarkably improve the photocatalytic degradation of methylene blue (MB) and RhB^39^.

Based on our literature survey, the photocatalytic applications of ZnO/WO_3_ nanocomposites for the metal removal/recovery from wastewater are still limited. Here, a set of ZnO/WO_3_ nanocomposites was hydrothermally synthesized and used as the photocatalyst for gold recovery from the industrial cyanide-based gold plating wastewater. Effects of various parameters were explored in order to find the best ZnO/WO_3_ photocatalyst and the best operating condition. A series of experimental results realized that the as-synthesized Z_5.0_/WO_3_ nanocomposite exhibited the highest photocatalytic activity to recover gold than the pristine semiconductors due to the synergetic effect of generated defects, a well-developed heterojunction and an appropriate alignment of band position with respect to the redox potential of active species. Besides, it possessed the excellent stability and reusability for the photocatalytic gold recovery.

## Experimental

*Synthesis of ZnO/WO*_*3*_* nanocomposites* A series of ZnO/WO_3_ nanocomposites was synthesized by a facile hydrothermal method adopted from Zaw et al.^40^. Briefly, a certain quantity of zinc nitrate 6-hydrate (Zn(NO_3_)_2_·6H_2_O, KemAus) was dissolved in 11.4 mL of *i*-propanol (*i*-C_3_H_7_OH, QRëC) and 50 mL of distilled (DI) water. Approximately 2 g of tungsten (VI) oxide (WO_3_, Sigma Aldrich) was subsequently added under the thorough stirring at 400 rpm at 25 °C for 4 h. Next, 1 mL of 3 M of hydrochloric acid (HCl, Merck) was gradually added. The slurry was stirred consecutively at the same stirring rate and temperature for 1 h. Subsequently, the obtained slurry was meticulously transferred into Teflon-lined stainless-steel autoclave and hydrothermally treated at 200 °C for 2 h. After a natural cooling, the solid portion was separated from the aqueous solution by centrifugation at 11,000 rpm (5804R, Eppendorf) for 10 min, washed several times by ethanol and DI water and finally dried overnight at 100 °C. The ready-to-use ZnO/WO_3_ nanocomposites at different weight contents of ZnO, denoted as Z_*x*_/WO_3_ (*x* is the weight percent of ZnO) were gained after calcination in air at 400 °C for 2 h.

*Characterization of ZnO/WO*_*3*_* nanocomposites* The morphologies and optical properties of synthesized ZnO/WO_3_ nanocomposites were analyzed as follows. The external microstructure and elemental distribution were observed by a field emission scanning electron microscopy (FE-SEM, JSM7610FPlus, JEOL) attached by an energy dispersive X-ray (EDS, ULTIM MAX 65) spectrometer. The interplanar spacing was monitored by a high-resolution transmission electron microscopy (TEM, JEM-3100F, JEOL) with an accelerating voltage of 300 kV. The crystallographic structure and local symmetry were evaluated by an X-Ray diffractometer (XRD, Bruker D2 Phaser) using Cu Kα X-ray and Raman spectroscopy (Perkin Elmer Spectrum GX). The Brunauer–Emmett–Teller (BET) surface area was examined by N_2_ adsorption/desorption according to the Barrett–Joyner–Halenda (BJH) technique at 77 K using a gas adsorption analyzer (Quantachrome® ASiQwin™). The surface element and electronic state was examined by an X-Ray photoelectron spectrometer (XPS, Axis Ultra, Kratos) with a delay line detector (DLD) and a monochromatic Al Kα (*hν* = 1486.6 eV) source. The optical absorption property was recorded by an UV visible near infrared spectrometer (Perkin Elmer Lambda 95). The behavior of photoinduced charge carriers was elucidated by a photoluminescence (PL) spectrometer (Perkin-Elmer LS-55) in air at 298 K with an excitation wavelength of 286 nm. The qualitative unpaired electron and defects were determined by an electron paramagnetic resonance spectroscopy (EPR, model, EMXmicro, Bruker) at 298 K.

*Photocatalytic activity of ZnO/WO*_*3*_* nanocomposites* The photocatalytic activity of all synthesized ZnO/WO_3_ nanocomposites was assessed for the gold recovery from the industrial cyanide-based gold plating wastewater collected from the circuit board manufacturing industry in Thailand. The concentrations of all metal ions were first measured by an inductive coupled plasma mass spectrometry (ICP-MS, PerkinElmer, NexION 2000). In each batch, the required quantity of explored photocatalyst was dispersed in 300 mL of the cyanide-based gold plating wastewater in cylindrical glass photoreactor having an inside diameter of 9 cm and height of 7 cm. The solid-wastewater mixture was thoroughly stirred at 400 rpm for 30 min in the absence of irradiated light to allow the uniform dispersion and adsorption of gold species on the photocatalyst surface. Subsequently, the reactor was irradiated by a high-pressure mercury lamp (400 W, 200–600 nm, RUV 533 BC). The distance between the reactor and light source was fixed at 28.5 cm, getting the light intensity of 3.57 mW/cm^2^. At particular time, approximate 5 mL of mixture was sampled and centrifuged at 11,000 rpm to separate the solid catalyst from the processed wastewater. The concentration of gold ions in the processed wastewater was analyzed by the flame atomic absorption spectrometry (Flame-AAS, Analyst 200 + flas 400; Perkin-Elmer). The percentages of gold recovery at particular time were estimated according to Eq. (1).

1 $$R (\mathrm{\%})\hspace{0.17em}=\hspace{0.17em}\left(\frac{{m}_{o}-{m}_{t}}{{m}_{0}}\right)\times 100$$

Where *R* is the gold recovery percentage, *m*_*o*_ is the initial mass of gold ions in wastewater and *m*_*t*_ is the mass of gold ions at time *t*.

## Results and discussion

The photocatalytic activity of all synthesized ZnO/WO_3_ nanocomposites was tested for gold recovery from the industrial cyanide-plating bath wastewater. The original wastewater depicted a weak basic property with the initial pH of 9.02–9.11. It contained gold ions at the concentration of 8–10 mg/L together with a trace quantity of copper ions (Cu^2+^), nickel ions (Ni^2+^), potassium ions (K^+^) and zinc ions (Zn^2+^) of less than 0.00006, 0.00047, 0.20285 and 2.27 mg/L, respectively.

### Characteristics of ZnO/WO_3_ nanocomposites

**Figure 1:**
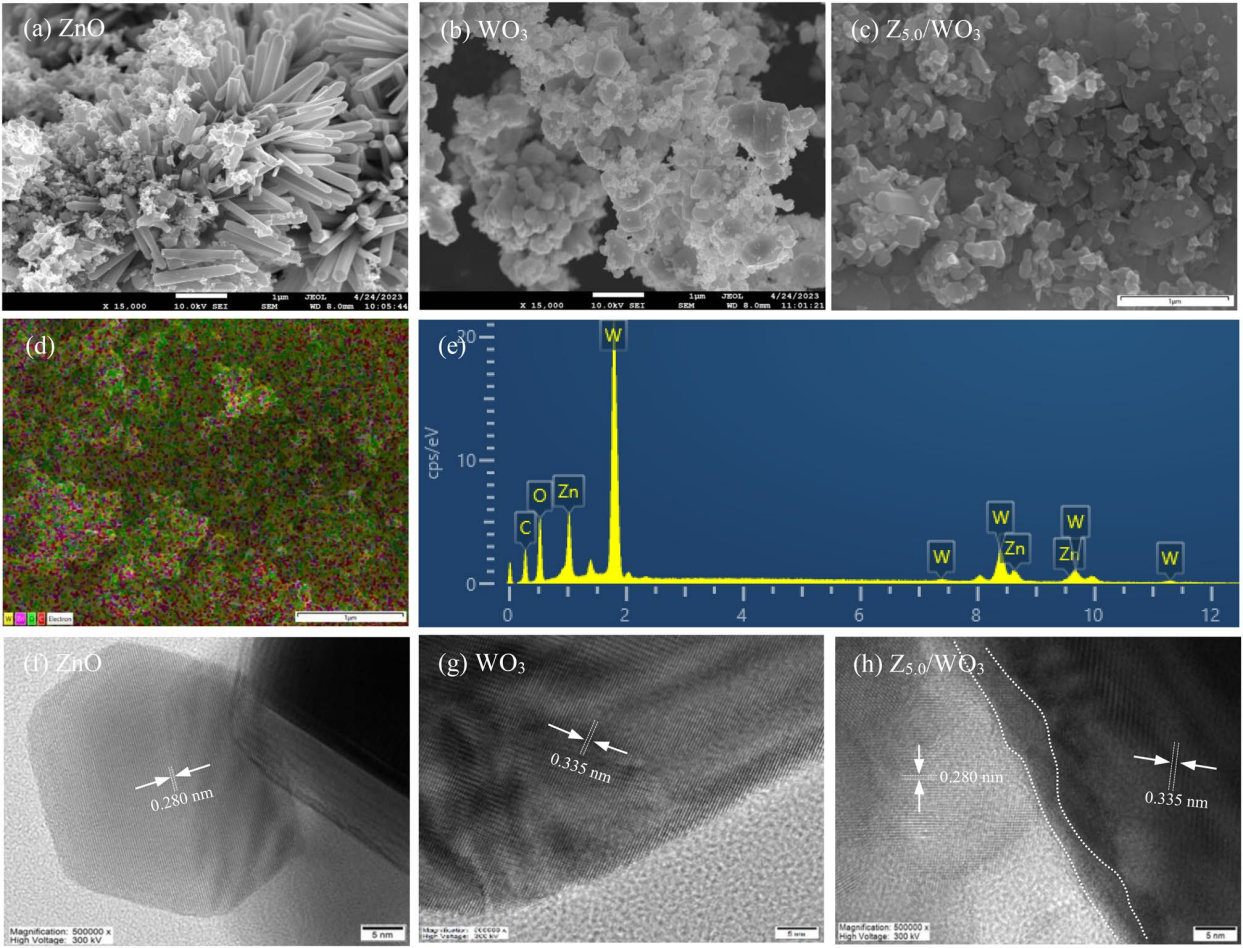
(**a**–**c**) SEM micrograph, (**d**) element mapping, (**e**) EDX spectrum and (**f**–**h**) high resolution TEM of (**f**) ZnO, (g) WO_3_ and (**h**) Z_5.0_/WO_3_ nanocomposite.

Regarding the external feature, the pure ZnO nanoparticles (NPs) exhibited a rod-like structure (Fig. 1a), while the pure WO_3_ NPs illustrated a flake-like structure (Fig. 1b). The Z_5.0_/WO_3_ nanocomposite revealed an agglomeration of WO_3_ surface (Fig. 1c). The presence of Zn, W and O elements was obviously detected with a good distribution (Fig. 1d and e). The high resolution TEM images of ZnO and WO_3_ illustrated the lattice stripes of 0.280 and 0.335 nm (Fig. 1f and g), relating to the (100) and (111) crystal planes of ZnO and WO_3_ NPs, respectively^33,35^. For the Z_5.0_/WO_3_ nanocomposite, a well-developed ZnO/WO_3_ composite was observed by the adherence of ZnO NPs on the surface of WO_3_ (Fig. 1h). The lattice fringes with *d* spacing of ZnO NPs (~ 0.280 nm) and WO_3_ NPs (~ 0.335 nm) were formed, confirming the presence of a tight contact between of ZnO-WO_3_ interfaces (dashed line) or the formation of the ZnO/WO_3_ heterostructure.

**Figure 2:**
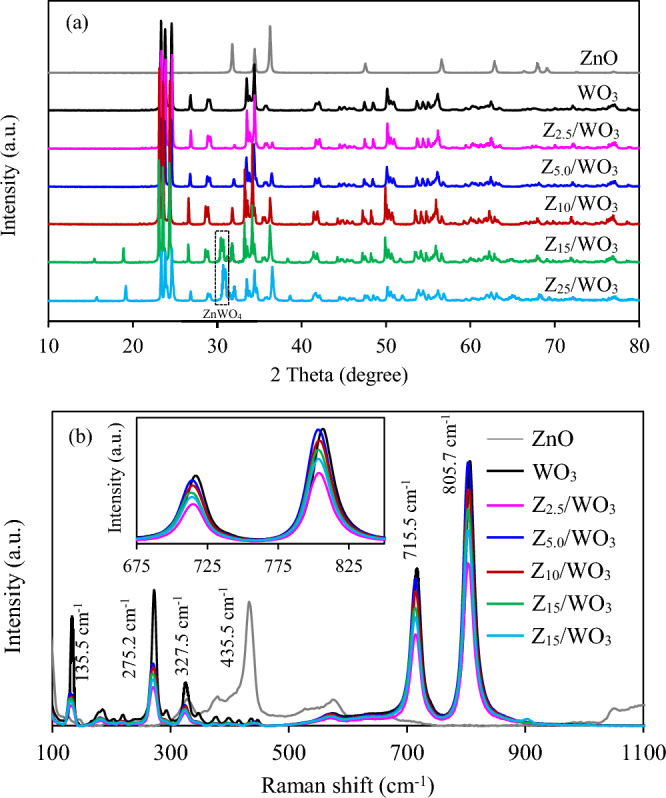
(**a**) XRD pattern and (**b**) Raman spectra of ZnO/WO_3_ nanocomposites.

Figure 2a depicts the XRD patterns of all synthesized ZnO/WO_3_ nanocomposites. A pure ZnO nanocomposite displayed the XRD peaks at 2θ of 31.8°, 34.4°, 36.3°, 47.6°, 56.5°, 62.9° and 67.9, indexing to the diffraction of the (100), (002), (101), (102), (110), (103) and (112) lattice planes of hexagonal wurtzite ZnO (JCPDS file no. 36-1451)^35^. The XRD peaks of WO_3_ sample were observed at 2θ values of 23.5°, 23.7°, 24.3°, 26.9°, 29.6°, 34.2°, 34.4°, 36.1°, 42.8°, 48.3°, 49.2°, 50.5° and 56.49°, arising from the diffraction of the (002), (020), (200), (120), (112), (022), (202), (-122), (222), (004), (040), (140) and (420) crystal planes of monoclinic WO_3_ (JCPDS file no. 43-1035)^33^. The XRD spectra of all synthesized ZnO/WO_3_ nanocomposites exhibited a predominate phase of monoclinic WO_3_, with a minor phase of hexagonal ZnO at 2θ of 31.8°, 36.3° and 56.5°. As the increase of ZnO contents, the intensity of the WO_3_ peak was relative weaker, while the ZnO peak was noticeably stronger. The distinct peaks of WO_3_ in ZnO/WO_3_ nanocomposites signified a homogeneous combination of both metal oxides^33^. In the presence of excessive ZnO contents (e.g. 15 and 25 wt%), additional peaks at 30.46°, 30.71° and 31.5° were observed, matching to the diffraction of (− 111), (111) and (020) lattice planes of monoclinic ZnWO_4_ (JCPDS file no. 15-0774)^41^.

To further confirm the crystal structures of ZnO/WO_3_ nanocomposites, the Raman spectroscopy was carried out. As shown in Fig. 2(b), the pure ZnO sample exhibited a sharp peak centered at 435.5 cm^−1^, arising from the oxygen-sub-lattice of hexagonal wurtzite ZnO sample^42^. The pure WO_3_ displayed the Raman spectrum at 135.5 cm^−1^ due to the lattice vibration, at 272.2 and 327.5 cm^−1^ due to the W–O–W bending mode of bridging oxide ions and at 715.5 and 805.7 cm^−1^ due to the W–O–W stretching mode^42–44^. All synthesized ZnO/WO_3_ nanocomposites illustrated a predominate Raman spectra of WO_3_ with different intensities. Typically, an intensive sharp Raman peak represents a high crystallinity of material^45^. Among all synthesized ZnO/WO_3_ nanocomposites, the Z_5.0_/WO_3_ nanocomposite exhibited the most intense and sharp Raman spectra, indicating its highest crystallinity. A blue shift of Raman spectra of all synthesized ZnO/WO_3_ was observed compared to those of pure WO_3_, indicating the existence of lattice defects^46^.

**Figure 3:**
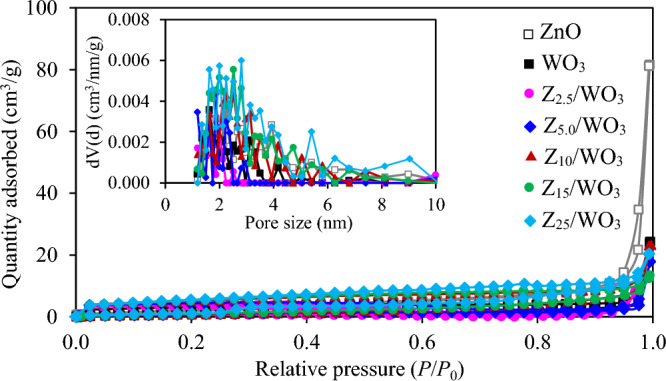
N_2_ adsorption/desorption isotherms of ZnO/WO_3_ nanocomposites.

As mentioned elsewhere, the surface properties of photocatalyst may affect the adoption of active molecule and subsequent charge transfer^16^, the surface area of all synthesized ZnO/WO nanocomposites was evaluated by the N_2_ adsorption/desorption isotherms. As demonstrated in Fig. 3, all samples exhibited the Type IV isotherm, which was the characteristic of mesoporous materials based on the recent IUPAC classification. A greatly increase of N_2_ adsorption volume at a relative pressure at 0.99 was attributed to the capillary condensation, which was a well index of a high homogeneity of synthesized samples. Quantitatively, the BET surfaces of pristine ZnO and WO_3_ were 17.8 and 8.8 m^2^/g, respectively, while those of Z_2.5_/WO_3_, Z_5.0_/WO_3_, Z_10_/WO_3_, Z_15_/WO_3_ and Z_25_/WO_3_ were 5.2, 7.9, 14.7, 17.3 and 22.0 m^2^/g, respectively. An increased BET surface area as the increased ZnO contents in nanocomposites might be caused by the increase of well-dispersed ZnO NPs on the WO_3_ surface.

**Figure 4:**
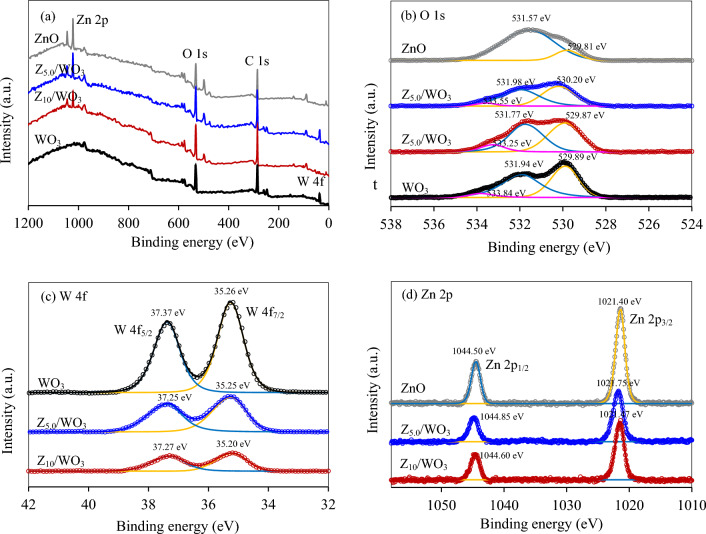
(**a**) Survey XPS and high resolution XPS spectra of (**b**) O 1*s*, (c) W 4*f* and (**d**) Zn 2*p* of Z_5.0_/WO_3_ and Z_10_/WO_3_ nanocomposites.

To explore the chemical surface composition and electronic state, the XPS analysis was carried out using C 1*s* peak of 284.6 eV as the reference position. As depicted in Fig. 4a, the survey XPS spectra of ZnO, WO_3_ and ZnO/WO_3_ nanocomposite showed the clear spectra of Zn 2*p*, O 1*s* and W 4*f* peaks and also the reference C 1*s* peak. For the O 1*s* XPS spectra (Fig. 4b), the ZnO possessed asymmetric broad peak, centered at ~ 531 eV (Fig. 4b). After deconvolution, two symmetric peaks appeared at 529.81 and 531.57 eV, corresponding to the Zn–O–Zn and surface –OH species^35,48^, respectively. The O 1*s* XPS spectra of WO_3_ were found at 529.89, 531.94 and 533.84 eV, belonging to the lattice oxygen, oxygen-absorbing H_2_O/adsorb oxides and surface –OH groups, respectively^49,50^. The O 1*s* XPS spectra of both nanocomposites displayed three principal peaks of lattice oxygens and adsorbed oxygens, which slight negative shift with respect to WO_3_. For the W 4*f* XPS spectra (Fig. 4c), two symmetric peaks at 35.26 and 37.37 eV were observed for WO_3_, respectively assigning to the W 4*f*_7/2_ and W 4*f*_5/2_ components, corresponding to the W^6+^ oxidation state^51^. Both Z_5.0_/WO_3_ and Z_10_/WO_3_ composites displayed the spectra of both W 4*f*_7/2_ and W 4*f*_5/2_ components with slight negative shifts by 0.01 and 0.12 eV and 0.06 and 0.10 eV compared with WO_3_, respectively. For the Zn 2*p* XPS spectra (Fig. 4d), the ZnO exhibited two XPS peaks at 1021.40 and 1044.50 eV, respectively assigning to the Zn 2*p*_3/2_ and Zn 2*p*_1/2_ components^52^. The positive shifts of either Zn 2*p*_1/2_ or Zn 2*p*_3/2_ elements were observed by 0.35 and 0.35 eV for Z_5.0_/WO_3_ and 0.07 and 0.10 for Z_10_/WO_3_. The positive shift of Zn elements and negative shift of W and O elements well indicated the interfacial electron transfer from ZnO to WO_3_ after hybridization^35,37^. Theoretically, the electron movements generally induce the formation of IEF at the ZnO-WO_3_ interface, which allows the transfer of electrons from WO_3_ to ZnO in the presence of light illumination according to the S-scheme mechanism^37,53,54^.

**Figure 5:**
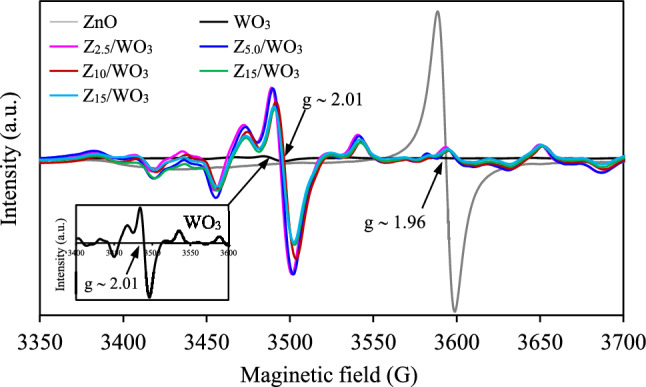
EPR spectra of ZnO/WO_3_ nanocomposites.

To investigate intrinsic defects in all synthesized samples, the EPR was conducted. Typically, the EPR signal ascribes the presence of unpaired electrons of the oxygen vacancies^50^. A high intensity of EPR signal indicates a high concentration of bulk oxygen vacancies^55,56^. As plotted in Fig. 5, the pristine ZnO displayed a very strong EPR signal at *g*-value of ~ 1.96, assigning to the singly ionized oxygen vacancy^57^. On the other hands, the pristine WO_3_ depicted a very weak EPR signal at *g*-value of ~ 2.01 (inset figure), suggesting a low defect quantity in its structure. Interestingly, all ZnO/WO_3_ nanocomposites exhibited strong EPR signals at *g*-value of ~ 2.01 indicating the formation of bulk oxygen vacancies in WO_3_ counterpart after hydrothermal and calcination, reasonably agreement with the Raman results. The highest EPR intensity was observed for the Z_2.5_/WO_3_ and Z_5.0_/WO_3_ nanocomposites, suggesting their comparable abundance of oxygen vacancies.

**Figure 6:**
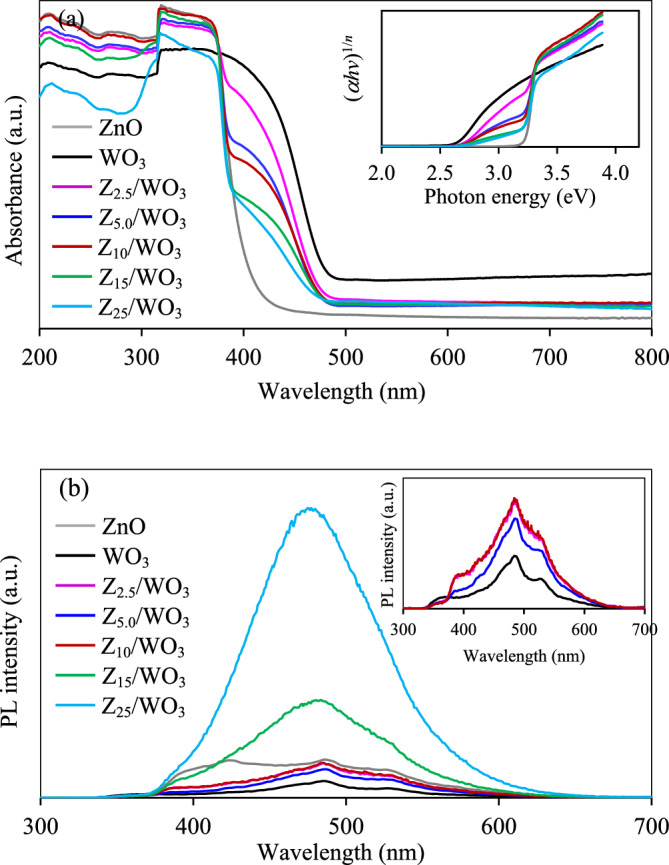
Representative (**a**) UV–Vis spectra with their Tauc plots (inset figure) and (**b**) PL spectra of different ZnO/WO_3_ nanocomposites.

To elucidate the energy level and light absorption performance, the UV–Vis near infrared spectroscopy was carried out. As presented in Fig. 6a, pure ZnO NPs possessed a strong absorbance at the wavelength below 400 nm, indexing an excellent UV light absorption performance, while pure WO_3_ NPs exhibited the absorption band edge at 480 nm, indicating an excellent absorption performance during the UV light and short-wave visible light. All synthesized ZnO/WO_3_ nanocomposites demonstrated the blueshift of absorption band edge with respect to the WO_3_ sample. The absorption intensity during the short-wave visible light decreased as the increase of ZnO contents. Via the use of Tauc equation (Eq. 2), plot of (*αhv*)^1/*n*^ and *hv* allowed to determine the value of bandgap (*E*_*g*_) by a linear extrapolation to the *x*-axis^58^.

2 $${(\alpha hv)}^\frac{1}{n}={A\left(hv-{E}_{g}\right)}$$

where *h* is Plank’s constant,* v* is the photon frequency, *α* is the absorption coefficient, *E*_*g*_ is the band gap, *A* is the proportional constant, and *n* is a factor related to the nature of the electron transition (½ for direct and 2 for indirect transition).

From Tauc plots, the pure ZnO and WO_3_ NPs possessed the bandgap energy of 3.24 and 2.66 eV, respectively and all synthesized ZnO/WO_3_ nanocomposites exhibited the bandgap between that of ZnO and WO_3_ counterparts. That is, the Z_2.5_/WO_3_, Z_5.0_/WO_3_ and Z_10_/WO_3_ nanocomposites possessed the similar bandgap of 2.69 eV, while the Z_15_/WO_3_ and Z_25_/WO_3_ nanocomposites depicted the bandgap of 2.71 and 2.73 eV, respectively. The VB edge and CB edge of pure ZnO and WO_3_ samples can be acquired from the following empirical equations (Eqs. 3–4)^29,35^, providing the *E*_VB_ of 2.92 and 3.43 eV and *E*_CB_ of − 0.32 and 0.77 eV for ZnO and WO_3_, respectively.

3 $$E_{{{\text{VB}}}} = X_{e} + 0.{5}E_{g} - E_{e}$$

4 $$E_{{{\text{CB}}}} = X_{e} - 0.{5}E_{g} - E_{e}$$

where *E*_CB_ and* E*_VB_ are the potential of CB and VB edges, respectively, *E*_*e*_ is the energy of free electron (4.5 eV) and *X*_*e*_ is the electronegativity of ZnO and WO_3_ (5.80 and 6.60, respectively^35^).

The qualitative recombination rate of photogenerated charge carriers of all synthesized ZnO/WO_3_ nanocomposites was explored via the PL spectrometer. Typically, the stronger the PL intensity, the higher the rate of charge recombination and vice versa^35^. For pure samples, as shown in Fig. 6b, the ZnO sample possessed the PL spectra during the UV and visible regions, and were higher than that of WO_3_, indicating a faster recombination rate of charge carriers. Among all ZnO/WO_3_ nanocomposites, the Z_5.0_/WO_3_ possessed the lowest PL intensity compared with other nanocomposites, suggesting its lowest recombination rate of charge carriers. This might be attributed to the presence of oxygen vacancies which can act as the electron trapping sites and extend the lifetime of electron–hole pairs as well as suppress the rate of charge recombination^35,59^.

## Photocatalytic activity of ZnO/WO_3_ nanocomposites

**Figure 7:**
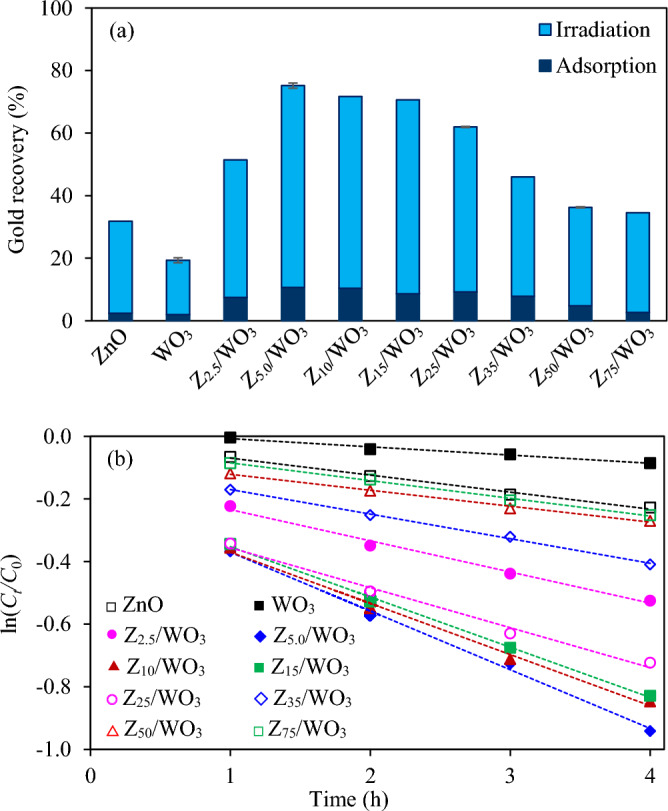
Effect of photocatalyst types on (**a**) photocatalytic gold recovery at irradiation time of 5 h and (**b**) linear variation of ln (*C*_*t*_/*C*_0_) versus time (*t*) using light intensity of 3.57 mW/cm^2^, catalyst dose of 2.0 g/L, 20 vol% C_2_H_5_OH and initial pH of wastewater of 9.11.

*Effect of coupled ZnO contents *The photocatalytic activity of all synthesized ZnO/WO_3_ nanocomposites was explored for gold recovery from the cyanide-based gold plating wastewater. As illustrated in Fig. 7a and Fig. S1, both pure ZnO and pure WO_3_ NPs exhibited a comparable adsorption capacity of gold ions (2.41 and 1.97% for ZnO and WO_3_, respectively). In the presence of irradiated light, the ZnO sample possessed a total photocatalytic activity for gold recovery up to 31.8%, approximately 1.6-time higher than that of WO_3_. A significant improved photocatalytic gold recovery was observed via the use of ZnO/WO_3_ nanocomposites. That is, total quantity of gold recovery was increased from 51.4 to 75.2% as the increase of ZnO content from 2.5 to 5.0 wt%. Further increasing the weight content of coupled ZnO to 75 wt% lessened the total quantity of gold recovery to 34.5%. The highest photocatalytic performance accounted from both adsorption (~ 10.7%) and photocatalytic reaction (~ 64.5%) was achieved at 75.2% via the use of Z_5.0_/WO_3_ nanocomposite. The rate of photocatalytic gold recovery was then fitted by the Langmuir–Hinshelwood model as expressed by Eq. (5)^60^. A plot of ln(*C*_*t*_/*C*_0_) versus reaction time *t* provides a negative slope, which allows to estimate the pseudo-first order reaction rate constant. Based on the model fitting (Fig. 7b), the Z_5.0_/WO_3_ nanocomposite possessed the highest value of pseudo first-order rate constant of 0.1876 h^−1^, approximately 3.4 and 7.1 times higher than that of ZnO and WO_3_, respectively.

5 $${\text{ln}}\left({C}_{t}/{C}_{0}\right)=-kt$$

where *C*_0_ is the concentration of gold ions at initial time, *C*_*t*_ is the concentration of gold ions at particular time* t* and *k* is the pseudo first-order rate constant.

Due to fact that the photocatalytic process is consisted of two sequential steps; adsorption and photocatalytic reaction, the adsorption experiment was also conducted for ZnO, WO_3_ and Z_5.0_/WO_3_ photocatalysts under the dark environment. As illustrated in Fig. S2, approximately 14.7, 11.2 and 15.31% of gold ions were removed by adsorption in the presence of ZnO, WO_3_ and Z_5.0_/WO_3_ photocatalysts, respectively. This suggested the main contribution of photocatalytic reaction for gold recovery compared with the adsorption.

As well known that the catalytic performance of photocatalyst is tightly related to the textural property, morphology, optical property and also the charge separation efficiency. Based on the obtained results, it seemed to be that the photocatalytic gold recovery of ZnO/WO_3_ photocatalysts did not depending on the crystal morphology and textural property. However, it can be presumed that the generated oxygen vacancies played a profound effect on the photocatalytic gold recovery of Z_5.0_/WO_3_ nanocomposite. This is because the generated oxygen vacancies can serve as the electron trapping sites, which can extend the lifetime of e^−^–h^+^ pairs and alleviate their recombination rate, which are beneficial for the photocatalytic performance. Besides, the heterojunction between WO_3_ and ZnO can act as the electron transfer bridge, which thereby extends the lifetime of charge separation as well as suppress the rate of charge recombination^35,37,61^.

**Figure 8:**
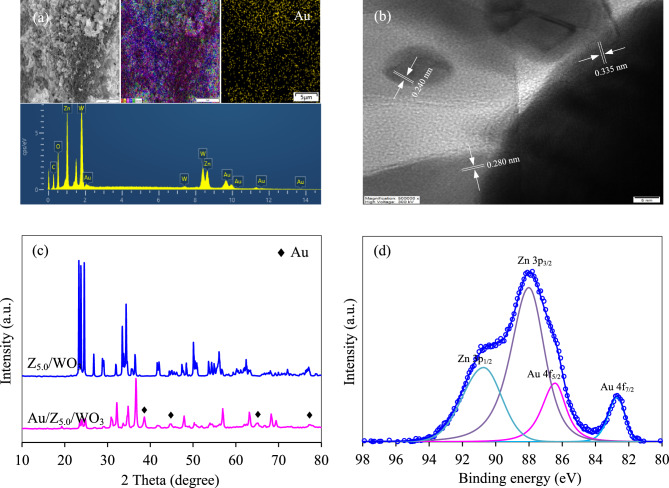
Representative (**a**) SEM–EDX diffractogram, (**b**) high resolution TEM images, (**c**) XRD pattern, (d) Au 4f. XPS spectra of Au/Z_5.0_/WO_3_ nanocomposite.

To confirm the presence of Au NPS deposited on the surface of ZnO/WO_3_ nanocomposites, the SEM–EDX was carried out. As depicted in Fig. 8a, the obtained Z_5.0_/WO_3_, denoted as Au/Z_0.5_/WO_3_ nanocomposite, displayed the rough surface with a uniform distribution of Au elements. A high resolution TEM image depicted clear lattice spacings of 0.280, 0.335 and 0.240 nm, indexing the (100), (111) and (111) crystal planes of ZnO, WO_3_ and Au, respectively (Fig. 8b). From the XRD analysis, apart from the concomitant phase of monoclinic WO_3_ and hexagonal ZnO, it revealed the main characteristic peaks at 2θ of 38.26, 44.47°, 64.72° and 77.74° (Fig. 8c), respectively corresponding to the (111), (200), (220) and (311) crystal plans of face-centered cubic Au NPs (PDF 01-077-9662). The Au 4*f* XPS spectra illustrated two broad peaks during the binding energy of 81–94 eV (Fig. 8d). After deconvolution, four peaks related to the Au 4*f*_7/2_ (82.73 eV), Au 4*f*_5/2_ (86.43 eV), Zn 3*p*_3/2_ (88.01 eV) and Zn 3*p*_1/2_ (90.73 eV) were observed. A spin–orbit splitting of ~ 3.7 eV indicated the presence of metallic gold^62^. The negative shift of Au 4*f* binding energy from usual values (84.0 and 87.7 for Au4*f*_7/2_ and Au 4*f*_5/2_, respectively), indicating the close contact and strong interaction of Au and ZnO^64^.

**Figure 9:**
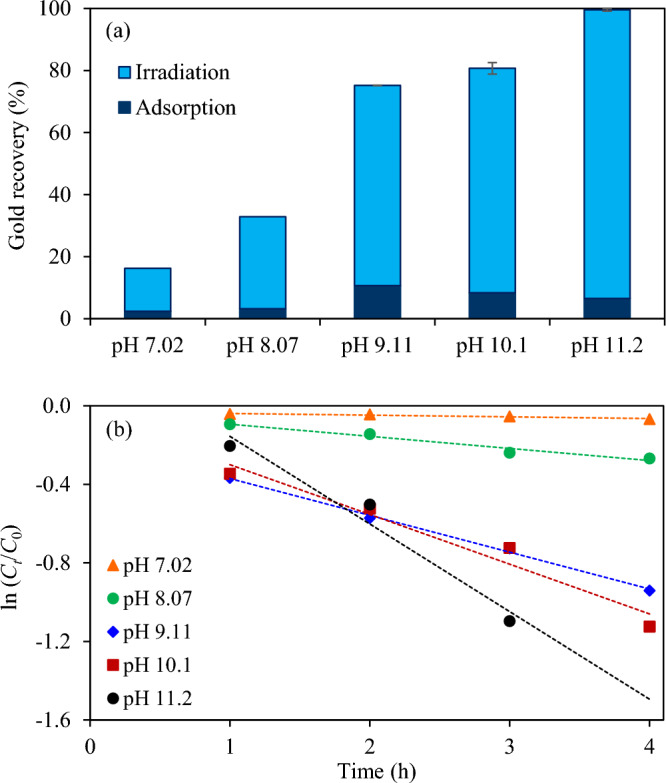
Effect of initial pH of wastewater on (**a**) photocatalytic gold recovery of Z_5.0_/WO_3_ nanocomposite at irradiation time of 5 h and (**b**) linear variation of ln (*C*_*t*_/*C*_0_) versus time (*t*) using light intensity of 3.57 mW/cm^2^, catalyst dose of 2.0 g/L and 20 vol% C_2_H_5_OH.

*Effect of initial pH of wastewater* The effect of initial pH of wastewater on the photocatalytic gold recovery was explored in the range of 7.02–11.2 via the Z_5.0_/WO_3_ nanocomposite. As displayed in Fig. 9a and Fig. S3, the quantity of adsorbed gold ions slightly increased as the increase of initial pH of wastewater from 7.02 to 9.11 and dropped afterward. The variation of ionic gold adsorption might be attributed to the interaction between the surface charges of photocatalyst and forms of gold cyanide complexes. That is, the semiconductors generally demonstrate positive surface charges when the pH is less than the zero-point charge pH (pH_ZPC_) and exhibit negative surface charges when the solution pH is more than the pH_zpc_ value^65^ and the gold cyanide complexes usually exhibit the stable form as [Au(CN)_2_]^−^ over a whole pH range at 25 °C and 1 atm^66^. In this case, the pH_zpc_ value of Z_5.0_/WO_3_ is ~ 8.0 (Fig. S4). A poor adsorption capacity at pH < pH_zpc_ was probably attributed to the positive charge repulsion between H^+^ to approach the W–OH surface ^67^. A high adsorption capacity at pH of 9.11 may be due to the formation of a weak repulsive force between the surface charges and the gold cyanide species, which still allowed an effective adsorption of active species on the surface of Z_5.0_/WO_3_ nanocomposite. However, a repulsive force became higher in a strong basic solution which can diminish the adsorption of active species. In the presence of irradiated light, the photocatalytic gold recovery increased as the increase of initial pH of wastewater, providing the pseudo first-order rate constants of 0.0087, 0.0168, 0.1876, 0.2535 and 0.4464 h^−1^ for pH 7.02, 8.07, 9.11, 10.1 and 11.2 respectively (Fig. 9b). A high photocatalytic gold recovery in a strong basic environment might be attributed to the fact that the contained OH^−^ species can effectively react with the photogenerated h^+^ to form the OH^·^ at VB, thus effectively suppressing the charge recombination as well as promoting the photocatalytic performance for gold recovery.

**Figure 10:**
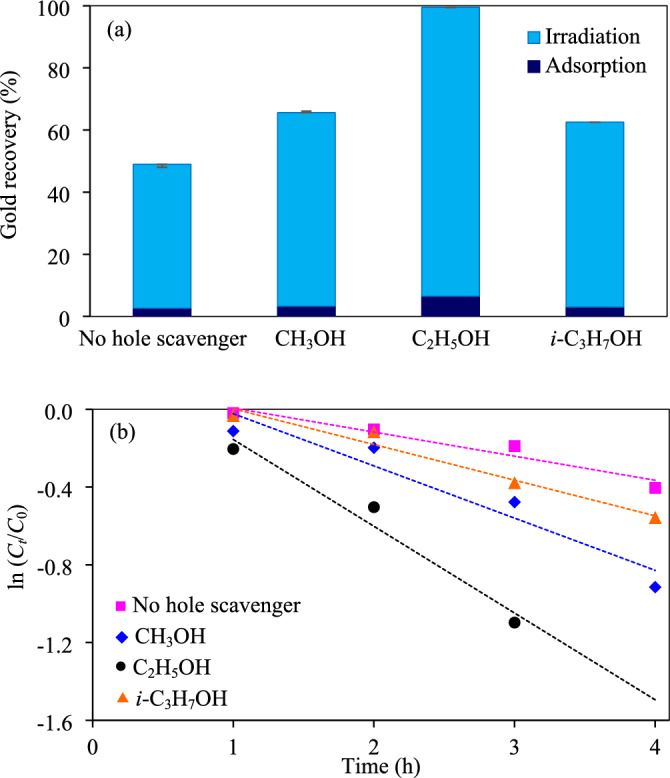
Effect of hole scavenger types on (a) photocatalytic gold recovery of Z_5.0_/WO_3_ nanocomposite at irradiation time of 5 h and (**b**) linear variation of ln (*C*_*t*_/*C*_0_) versus time (*t*) using light intensity of 3.57 mW/cm^2^, catalyst dose of 2.0 g/L, hole scavenger concentration of 20 vol% and initial pH of wastewater of 11.2.

*Effect of hole scavenger types and concentrations* As mentioned elsewhere, the addition of hole scavengers can improve the photocatalytic metal removal by the hole interception, thus allowing the photogenerated electrons to bound the adsorbed gold ions to proceed the photocatalytic reaction^69^. In this part, the effect of different hole scavengers including methanol (CH_3_OH), ethanol (C_2_H_5_OH) and isopropanol (*i*-C_3_H_7_OH) on the photocatalytic gold recovery was explored via the Z_5.0_/WO_3_ nanocomposite. As shown in Fig. 10a and Fig. S5, approximately 49% of gold ions was recovered within 5 h in the absence of hole scavenger. An improved photocatalytic activity for gold recovery was obviously observed in the presence of hole scavengers. The gold recovery percentages increased as the order of ethanol > methanol > *i*-propanol > no hole scavenger, providing the pseudo first-order rate constants of 0.4464, 0.2687, 0.1835 and 0.1240 h^−1^, respectively (Fig. 10b), probably attributed to the effect of different oxidation potentials of each hole scavenger. That is, the lower the oxidation potential, the better the hole scavenger performance^70,71^. On the basis of different oxidation potentials; methanol (0.016 V/NHE^71^), ethanol (0.084 V/NHE^71^, *i*-propanol (0.105 V/NHE^71^), the methanol-contained system should illustrate the highest photocatalytic gold recovery. Bases on the obtained results, the exception from the trend indicated that there are some other factors that may play a crucial role on the photocatalytic gold recovery. A low photocatalytic activity of methanol might be attributed to a strong surface adsorption of formed intermediate species. Based on the find out of Santasalo-Aarnio et al.^72^, both formate and bonded CO were the main intermediate species generated in the methanol oxidation, which can adsorb and block the active sites of photocatalyst to complete the photocatalytic reaction^71^. In the presence of ethanol, it can react with the photogenerated h^+^, yielding C_2_H_4_O as the reaction product^73^.

**Figure 11:**
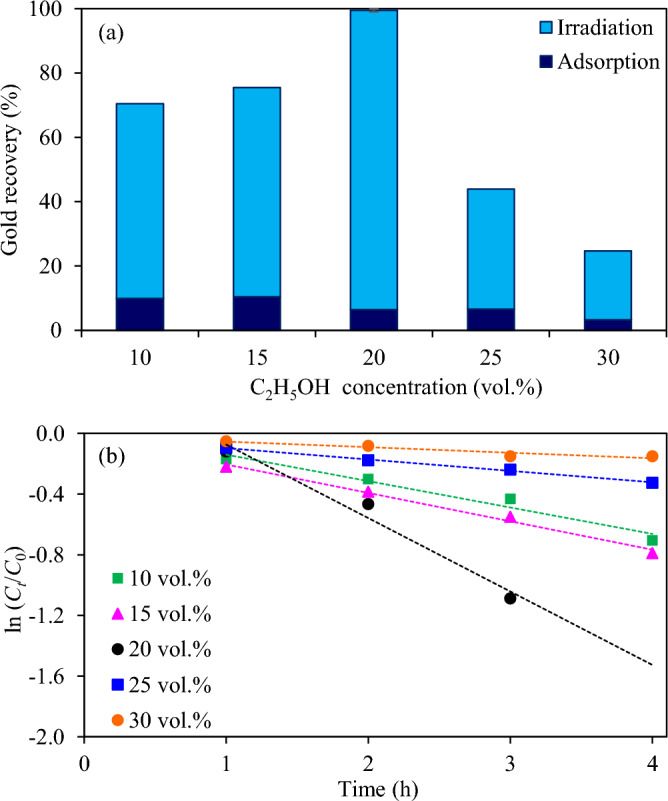
Effect of ethanol concentrations on (**a**) photocatalytic gold recovery of Z_5.0_/WO_3_ nanocomposite at 5 h and (**b**) linear variation of ln (*C*_*t*_/*C*_0_) versus time (*t*) using light intensity of 3.57 mW/cm^2^, catalyst dose of 2.0 g/L and initial pH of wastewater of 11.2.

Figure 11a and Fig. S6 illustrated the effect of ethanol concentrations in the range of 10–30 vol% on the photocatalytic gold recovery. It is noteworthy that the increase of ethanol concentrations from 10 to 20 vol% led to the increase of gold recovery from 70.4 to 99.5%. However, further increasing the ethanol concentration to 30 vol% diminished the gold recovery. In terms of pseudo first-order rate constants, they were 0.1741, 0.1874, 0.4464, 0.0751 and 0.0367 h^−1^ at ethanol concentrations of 10, 15, 20, 25 and 30 vol%, respectively (Fig. 11b). A poor performance of gold recovery at low ethanol concentration was probably attributed to a fast recombination rate of charge carriers due to the limitation of hole scavenger quantity. In the presence of high ethanol concentration, excess ethanol molecules may compete to each other or other active molecules to complete the oxidation reaction^74^ and probably hinder the adsorption of other reactive species on active sites, which in turn reduced the photocatalytic performance. A poor photocatalytic activity in the presence of inappropriate content of hole scavengers was also reported for other photocatalytic systems^74–76^.

**Figure 12:**
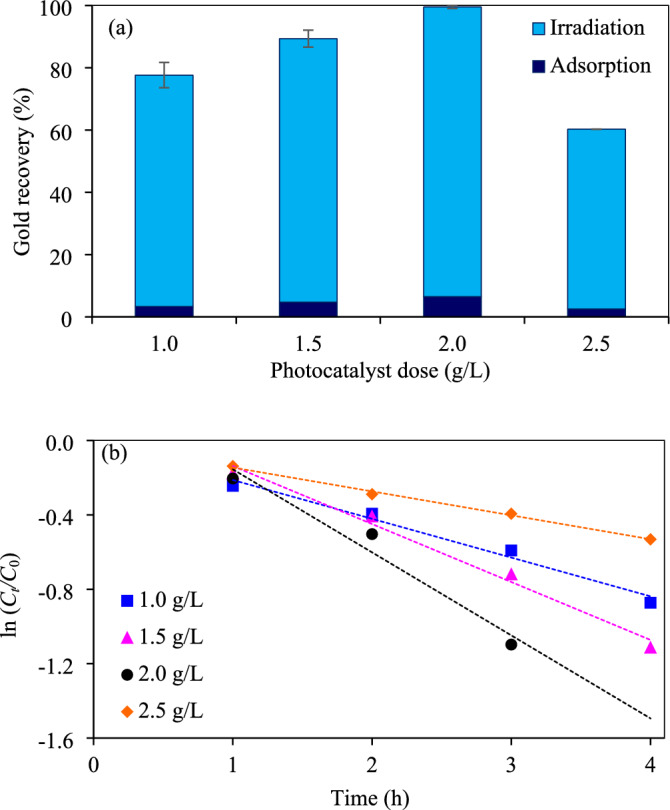
Effect of Z_5.0_/WO_3_ nanocomposite doses on (**a**) photocatalytic gold recovery at 5 h and (**b**) linear variation of ln (*C*_*t*_/*C*_0_) versus time (*t*) using light intensity of 3.57 mW/cm^2^, ethanol concentration of 20 vol% and initial pH of wastewater of 11.2.

*Effect of photocatalyst doses* The effect of photocatalyst doses on the photocatalytic gold recovery was also explored as depicted in Fig. 12a and Fig. S7. It is worth noting that the gold recovery increased from 77.6 to 99.5% as the increase of photocatalyst doses from 1.0 to 2.0 g/L. However, further rising the photocatalyst dose to 2.5 g/L decreased the photocatalytic performance. The system with the photocatalyst dose of 2.0 g/L exhibited the pseudo first-order  rate constant of 0.4464 h^−1^, approximately 2.1, 1.4 and 3.5 times higher than that of 1.0, 1.5 and 2.5 g/L, respectively (Fig. 12b). Typically, a high photocatalyst dose usually exhibits a high active surface area and electrons, which can facilitate an effective photocatalytic reaction^76^. However, too high photocatalyst dose induced a high suspension turbidity, consequently causing a low light penetration and light scattering^77^. In addition, a high photocatalyst dose usually conducted a non-homogeneous suspension because of the photocatalyst agglomeration^78^.

The comparative efficacy of gold recovery from gold-cyanide complexes was finally conducted between the as-synthesized Z_5.0_/WO_3_ nanocomposite and other photocatalysts. As shown in Table 1, different works depicted different percentages of photocatalytic gold recovery, attributing to their different utilized matrix/wastewater as well as operating conditions to test the photocatalytic performance. The photocatalytic performance of Z_5.0_/WO_3_ is seemed to be lower than ZnO, diagnosing from the use of longer operating time with low initial concentration of gold ions. Via rGO/TiO_2_ photocatalyst, it is hard to compare the between them because they used the gold ions with high concentration (300 mg/L). Under almost similar working conditions, the photocatalytic performance of Z_5.0_/WO_3_ nanocomposite was comparable to TiO_2_/WO_3_, but lower than that of Au/TiO_2_.

**Table 1:** Comparative efficacy of gold recovery from gold-cyanide complexes by photocatalytic process.

Photocatalysts	Synthesis method	Matrix/wastewater		Operating conditions			Gold recovery	Ref
		Initial conc. of gold ions (mg/L)	pH	Light source (Intensity)	Catalyst loading (g/L)	Hole scavenger		
ZnO	Solution combustion	60	n.d.*	UV light	3	10 vol% CH_3_OH	100% (0.5 h)	^21^
rGO/TiO_2_ (1 wt% rGO)	Photoreduction	300	n.d	LuzChem photoreactor (6 lamps, 365 nm)	n.d	500 mL i-C_3_H_8_O	~ 30% (3 h)	^23^
Au/TiO_2_ (0.5 wt% Au)	Photodeposition	15	8.4–9.2	High pressure Hg lamp (3.20 mW/cm^2^)	1	20 vol% CH_3_OH	100% (0.5 h)	^24^
TiO_2_/WO_3_ (60 wt%)	Hydrothermal	10–15	8.4–9.2	High pressure Hg lamp (3.20 mW/cm^2^)	2	10 mM Na_2_S_2_O_3_ and 20 vol% C_2_H_5_OH	98.2% (5 h)	^80^
ZnO/WO_3_ (5 wt% ZnO)	Hydrothermal	8–10	11.2	High pressure Hg lamp (3.57 mW/cm^2^)	2	20 vol% C_2_H_5_OH	99.5% (5 h)	This work

*Not detected.

**Scheme 1:**
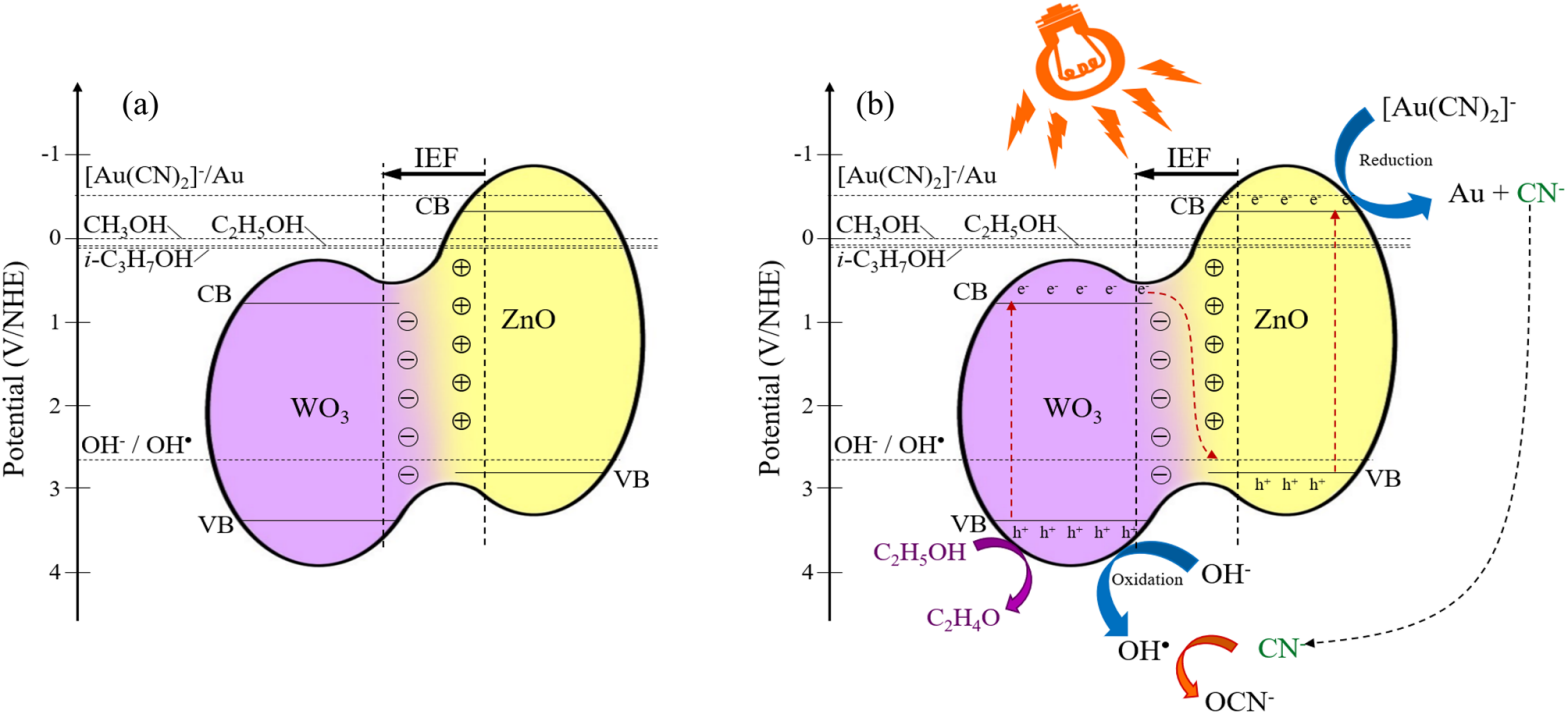
Proposed mechanism of photocatalytic gold recovery from the cyanide-based gold plating wastewater via ZnO/WO_3_ nanocomposites in the (**a**) absence and (**b**) presence of UV–Vis irradiation.

*Mechanism of gold recovery by ZnO/WO*_*3*_* nanocomposites* The reaction mechanism of photocatalytic gold recovery from the cyanide-based gold plating wastewater via ZnO/WO_3_ nanocomposites was roughly proposed on the basis of obtained results and literature. According to the density functional theory (DFT) calculation of He et al.^35^, the heterojunction of ZnO and WO_3_ induced the positive electric surface on ZnO and negative electric surface on WO_3_, thus forming the p–n junction where ZnO and WO_3_ were the n-type and p-type, respectively. Via this junction, the electrons can transfer from ZnO to WO_3_, while the holes can migrate from WO_3_ to ZnO after hybridization^28,33,35,79^. In this work, the transfer of electrons from ZnO to WO_3_ was clearly evidenced by a positive shift of Zn 2*p* XPS spectra and a negative shift of W 4*f* XPS and O 1*s* XPS spectra (Fig. 4b–d). Based on the calculated *E*_VB_ and *E*_CB_ of ZnO and WO_3_ according to Eqs. (3)-(4), the ZnO had a more negative value of *E*_CB_ than that of WO_3_ (− 0.32 and 0.77 eV for ZnO and WO_3_, respectively). According to a low Fermi energy levels of WO_3_ (− 4.52 eV^37^) compared with ZnO (− 4.29 eV^37^), the electrons can certainly transfer from ZnO to WO_3_ after heterojunction to equilibrate the Fermi levels of both counterparts (Scheme 1a). Such electron movements induced the build-up of IEF at the ZnO–WO_3_ interface directing from ZnO to WO_3_^35,37^. In the presence of irradiation, the generated IEF can accelerate the recombination of weak redox power of electrons at CB of WO_3_ and holes at VB of ZnO according to the S-scheme mechanism^37,53,54^, thus allowing a spatial separation of strong redox power of electrons at CB of ZnO and holes at VB of WO_3_ to proceed the photocatalytic reaction. Therefore, the photoreduction of [Au(CN)_2_]^−^ to metallic gold (reaction (R2)) occurred at the CB of ZnO, while photooxidation of OH^−^ (reactions (R3)-(R4)) and C_2_H_5_OH emerged at the VB of WO_3_ as proposed in Scheme 1b. This statement was clearly confirmed by a high photocatalytic gold recovery of ZnO compared with that of WO_3_ (Fig. 7).

**Figure 13:**
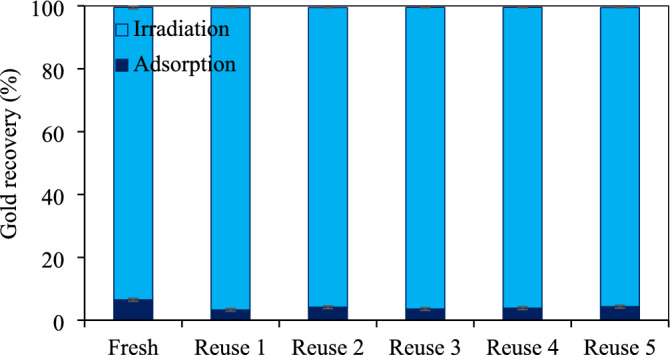
Reusability of Z_5.0_/WO_3_ nanocomposite for gold recovery at 5 h using light intensity of 3.27 mW/cm^2^, catalyst dose of 2.0 g/L, ethanol concentration of 20 vol% and initial pH of wastewater of 11.2.

*Reusability of ZnO/WO*_3_ *nanocomposites* The reusability of Z_5.0_/WO_3_ nanocomposite was also examined for the photocatalytic gold recovery for several consecutive runs. As demonstrated in Fig. 13, no noticeable change of the photocatalytic gold recovery was observed even at the 5th reuse (99.4%). This indicated a high stability and reusability of the hydrothermally synthesized ZnO/WO_3_ nanocomposites. The resultant Au decorated ZnO/WO_3_ (Au/ZnO/WO_3_) obtained from this work may be used for other applications such as desulfurization of thiophene^36^, H_2_O_2_ production^37^, or gas sensing^41^. However, the application of resultant Au/ZnO/WO_3_ was not conducted in the work due to it beyonds the scope.

## Conclusions

A set of ZnO/WO_3_ nanocomposites was facile synthesized by hydrothermal method for photocatalytic gold recovery from the industrial cyanide-based gold plating wastewater. An appropriate content of ZnO NPs in the ZnO/WO_3_ nanocomposites was first explored and subsequently followed by the investigation of optimum operating condition. The experimental results demonstrated that the ZnO/WO_3_ nanocomposites exhibited a higher photocatalytic gold recovery than both pristine counterparts. The Z_5.0_/WO_3_ nanocomposite possessed the highest photocatalytic activity for gold recovery due to the synergetic effect of oxygen vacancies and the formed S-scheme heterojunction, which can serve as electron trapping sites to extend the lifetime of electron–hole pairs and suppress the combination rate of charge carriers. Besides, appropriate band position alignment of nanocomposite with respect to the redox potential of gold-cyanide species encouraged the reduction of [Au(CN)_2_]^−^ at CB of ZnO and the oxidation of hole scavengers at VB of WO_3_. Via the Z_5.0_/WO_3_ nanocomposite, approximately 99.5% of  gold ions was recovered within 5 h using the light intensity of 3.57 mW/cm^2^, catalyst dose of 2.0 g/L, ethanol concentration of 20 vol% and initial pH of wastewater of 11.2. In addition, it possessed a high stability and reusability even at the 5th reuse. This work may shade the light for the design and application of ZnO/WO_3_ nanocomposites for precious metal recovery from a real industrial wastewater.

### Supplementary Information

Supplementary Figures.

## Data Availability

All data generated or analyzed during this study are included in this published article.
