# Supplementary material for: Highly efficient ZnO/WO3 nanocomposites towards photocatalytic gold recovery from industrial cyanide-based gold plating wastewater
Source: Sci Rep. 2023 Dec 20;13:22752. doi: 10.1038/s41598-023-49982-6 (PMC10733279; doi:10.1038/s41598-023-49982-6)
Supplement: Supplementary file 1 — Supplementary Figures. [file 41598_2023_49982_MOESM1_ESM.docx]

**Supporting document**

**Highly efficient ZnO/WO_3_ nanocomposites towards photocatalytic gold recovery
from industrial cyanide-based gold plating wastewater**

Satjaporn Sangkhanak^a,b^, Naphaphan Kunthakudee^b^, Mali Hunsom^b,c,^^[[1]](#footnote-1)^*, Prakorn Ramakul^d^,
Karn Serivalsatit^e,d^, Kejvalee Pruksathorn^a^

^a^Department of Chemical Technology, Faculty of Science, Chulalongkorn University, Phayathai Road, Pathumwan, Bangkok 10330, Thailand

^b^Department of Chemical Engineering, Faculty of Engineering, Mahidol University,
Phuttamonthon 4 Road, Nakhon Pathom, 73170, Thailand

^c^Associate Fellow of Royal Society of Thailand (AFRST), Bangkok 10300 Thailand

^d^Department of Chemical Engineering, Faculty of Engineering and Industrial Technology,
Silpakorn University, Nakhon Pathom 73000, Thailand

^e^Department of Materials Science, Faculty of Science, Chulalongkorn University, Phayathai Road, Pathumwan, Bangkok 10330, Thailand

^e^Photocatalysts for Clean Environment and Energy Research Unit, Faculty of Science, Chulalongkorn University, Bangkok 10330, Thailand

Figure S1. Variation of *C_t_*/*C*_0_ against time of different Z*_x_*/WO_3_ for photocatalytic gold recovery at irradiation time of 5 h using light intensity of 3.57 mW/cm^2^, catalyst dose of 2.0 g/L, 20 vol.% C_2_H_5_OH and initial pH of wastewater of 9.11.

**□** ZnO

◼ WO_3_

**⯁** Z_5.0_/WO_3_

Figure S2. Variation of *C_t_*/*C*_0_ against time of ZnO, WO_3_ and Z_5.0_/WO_3_ for photocatalytic gold recovery at dark condition for 5 h, catalyst dose of 2.0 g/L, 20 vol.% C_2_H_5_OH and initial pH of wastewater of 9.11.

Figure S3. Variation of *C_t_*/*C*_0_ against time of Z_5.0_/WO_3_ at different pH for photocatalytic gold recovery at irradiation time of 5 h using light intensity of 3.57 mW/cm^2^, catalyst dose of 2.0 g/L and 20 vol.% C_2_H_5_OH

Figure S4. Determination of pHzpc of Z_5.0_/WO_3_

Figure S5. Variation of *C_t_*/*C*_0_ against time of Z_5.0_/WO_3_ at different types of hole scavenger for photocatalytic gold recovery at irradiation time of 5 h using light intensity of 3.57 mW/cm^2^, catalyst dose of 2.0 g/L, hole scavenger concentration of 20 vol.% and initial pH of wastewater of 11.2.

Figure S6. Variation of *C_t_*/*C*_0_ against time of Z_5.0_/WO_3_ at different ethanol concentrations for photocatalytic gold recovery at irradiation time of 5 h using light intensity of 3.57 mW/cm^2^, catalyst dose of 2.0 g/L and initial pH of wastewater of 11.2.

Figure S7. Variation of *C*/*C*_0_ against time of Z_5.0_/WO_3_ at different photocatalyst doses for photocatalytic gold recovery at irradiation time of 5 h using light intensity of 3.57 mW/cm^2^, ethanol concentration of 20 vol.% and initial pH of wastewater of 11.2.

1. * Corresponding author: E-mail address: mali.hun@mahidol.edu [↑](#footnote-ref-1)
